# Human papillomavirus self‐collection: The long road from scientific evaluation to implementation in screening programs

**DOI:** 10.3322/caac.70047

**Published:** 2025-12-04

**Authors:** Nicolas Wentzensen, Armando Baena

**Affiliations:** ^1^ Division of Cancer Epidemiology and Genetics National Cancer Institute, National Institutes of Health Rockville Maryland USA

The understanding that persistent infections with human papillomavirus (HPV) cause nearly all cervical cancers has led to important cervical cancer prevention approaches: HPV vaccination is highly efficacious at preventing HPV infection when administered before infection occurs. In cervical cancer screening, HPV testing has higher sensitivity, provides longer reassurance when a test is negative, and has an overall better tradeoff of benefits and harms compared with cytology.^1^ Consequently, HPV testing is gradually replacing cytology as a primary screening test in screening programs worldwide.

An additional benefit of HPV testing is that it can be conducted from self‐collected vaginal specimens, with high concordance to clinician collected specimens. The article by Perkins et al.^2^ describes the adoption of clinical guidelines for HPV self‐collection developed by the Enduring Guidelines effort^3^, ^4^ for the American Cancer Society cervical cancer screening guideline.^5^

Using self‐collected specimens for cervical cancer screening has great promise to expand screening to unscreened and underscreened populations who may not have access to clinician‐based sampling or who may decide not to participate in screening to avoid pelvic examinations.

HPV self‐collection is not a new development—the first studies evaluating self‐collection were conducted in the 1990s and focused on different sampling strategies, including vaginal lavages, tampon collections, and vaginal brush devices.^6^, ^7^, ^8^, ^9^ Of these, vaginal sampling devices were the most successful and have become the standard for HPV self‐collection today. More recently, urine‐based HPV testing has been evaluated as a possible alternative to clinician and brush‐based self‐collection, with heterogeneous efficacy results to date.^10^

## CLINICAL AND REGULATORY EVALUATION OF HPV SELF‐COLLECTION

Since the initial proof‐of‐concept studies, HPV self‐collection has been evaluated in many, mostly cross‐sectional studies, as summarized in a series of systematic reviews.^11^, ^12^, ^13^, ^14^ The systematic reviews demonstrate that polymerase chain reaction (PCR)‐based HPV tests have high agreement for detection of HPV and cervical precancer between clinician‐collected and self‐collected specimens. They have also demonstrated the high acceptability of HPV self‐collection, with several studies indicating that screening participants prefer self‐collection over clinician‐collection. There is strong evidence that high sensitivity for precancer detection using self‐collected samples is only achieved with PCR‐based HPV DNA tests, as opposed to signal amplification or messenger RNA‐based tests, which are not considered equivalent. Several components need to be considered when evaluating HPV self‐collection, including the type of HPV assay, the type of collection device, the type of storage until processing (e.g., dry storage vs. liquid storage), and the sample dilution at the time of transfer to a liquid buffer before testing. Each of these factors may affect performance and has to be evaluated in the context of the proposed self‐collection approach. The regulatory evaluation of HPV self‐collection, which is a requirement for clinical use in the United States, considers specific combinations of tests, devices, buffers, and processing approaches rather than providing blanket approval for HPV self‐collection. To date, all evaluations for regulatory approval were based on concordance of HPV detection, not identification of precancers. Two different regulatory pathways have been used for self‐collection in the United States. Initially, self‐collection approaches using specific devices were evaluated as extended indications for HPV tests already approved for primary HPV screening, leading to approval for self‐collection in a clinical setting.^3^ Recently, a new self‐collection device for home collection was approved specifically for use with an HPV assay that was previously approved for primary HPV screening.^15^ Clinical and regulatory evaluation of other home‐collection approaches is currently underway. To add to the confusion, several HPV self‐collection services are offered that are not regulated because they are classified as laboratory‐developed tests. These sampling approaches and tests may be insufficiently validated and are not covered by current clinical recommendations. It can be difficult for clinicians and patients to distinguish self‐collection approaches with regulatory approval from these unregulated, laboratory‐developed tests.

## IMPLEMENTATION OF HPV SELF‐COLLECTION IN SCREENING PROGRAMS

The Netherlands and Australia were the first countries to include HPV self‐collection in their organized screening programs. The experience of implementing HPV self‐collection in these screening programs has taught us many invaluable lessons. For example, an early evaluation of the Dutch program demonstrated lower relative sensitivity of self‐collection compared with clinician collection for the detection of cervical precancer, which led to adjusting the resuspension volume of self‐collected specimens.^16^ In both programs, the initial goal was to offer self‐collection to those individuals who do not regularly participate in screening. However, because these opt‐in models faced challenges of patient and provider education and implementation, both programs now offer HPV self‐collection to all screening participants. In the Netherlands, all women aged 30 years and older receive a self‐sampling kit by mail, allowing them to perform the test at home.^17^ Those who have a positive HPV result are subsequently invited to their general practitioner for triage with cytology. In Australia, women aged 25–74 years can obtain a self‐collection kit through a health care provider.^18^ Those who test positive for HPV types 16/18 are referred directly to colposcopy, whereas those who test positive for other oncogenic HPV types undergo triage with cytology, followed by colposcopy if cytology is abnormal. The program integrates self‐collection within existing screening and follow‐up pathways to ensure timely clinical management.

In the United States, regulatory approval and clinical recommendations state that clinician collection is preferred, but self‐collection is acceptable, which allows wide use of HPV self‐collection. Because the United States does not have a nationally organized screening program, implementation of HPV self‐collection will vary locally and regionally. Adoption in some integrated health care systems, as well as state‐funded and federally funded screening programs, is currently underway.

Many low‐to‐middle income countries are turning to HPV self‐collection to close their gaps in cervical cancer screening coverage. Countries such as Argentina, Chile, Colombia, Mexico, Peru, and several others across Africa and Asia have either included self‐collection in their national guidelines or have started pilot programs, mainly targeting underscreened or never‐screened women aged 30–65 years.^19^ Generally, self‐collection in low‐to‐middle income countries focuses on high‐risk and hard‐to‐reach populations, leveraging existing cytology‐based infrastructure, community health workers, and established follow‐up systems.

Concerns have been raised by some clinicians that self‐collection foregoes the clinical evaluation and pelvic examination required for clinician sampling, missing an opportunity for a pelvic examination that may lead to the detection of other health problems. However, the value of pelvic examinations beyond obtaining a cervical cancer screening sample in an average‐risk individual is not clear. Another concern that has been raised is that HPV self‐collection may be offered as an over‐the‐counter test, possibly leading to unnecessary and unindicated testing with unclear consequences. However, regulatory authorities and clinical guidelines have made it clear that HPV self‐collection is prescription‐based, requiring a provider to order the test and to follow‐up with patients to communicate test results and recommend subsequent management.

## IMPORTANT DIFFERENCES BETWEEN CLINICIAN COLLECTION AND SELF‐COLLECTION: EVIDENCE GAPS

Despite many similarities, there are some important differences between self‐collection and clinician collection that affect how self‐collection can be currently implemented. First, there is currently no validated and approved test for triage that can be conducted using self‐collected specimens, beyond limited or extended genotyping, which is provided by some HPV tests. Currently approved triage tests either have insufficient accuracy (cytology) or are not sufficiently evaluated (dual stain) in self‐collected specimens. There are some promising data from studies of methylation markers; however, to date, no methylation assay has been sufficiently validated and approved in the United States. Developing a reliable triage approach for self‐collected specimens is a high priority for research and development. Consequently, among those with positive screening results, an additional clinician‐collected sample is required that enables conducting currently approved triage tests.^3^ This may require an additional visit or additional sample collection at the colposcopy visit for those who are referred to colposcopy after testing positive for HPV16/18 following self‐collection.

Most self‐collection studies have been cross‐sectional, with few including short‐term follow‐up. Long‐term studies are missing to inform the reassurance after a negative HPV test from a self‐collected specimen. Although extrapolating from cross‐sectional data to long‐term reassurance has become widely accepted for the evaluation of new PCR‐based HPV tests in clinician‐collected samples, it is not clear whether we can extrapolate in the same way for self‐collected specimens because different sites may be sampled. Therefore, current guidelines recommend a 3‐year screening interval for those who test negative in HPV self‐collection samples compared with those who test negative in a clinician‐collected specimen, for which 5‐year intervals are recommended. A self‐collection study with long‐term follow‐up is currently underway and will soon provide 5‐year follow‐up data after negative HPV results in self‐collected specimens,^20^ allowing extended intervals if long‐term reassurance is comparable between HPV self‐collection and clinician collection.

## SUMMARY

Self‐collection has come a long way from initial evaluation to regulatory approval and inclusion in clinical guidelines in the United States. We applaud the decision of the American Cancer Society Guideline Development Group to adopt the HPV self‐collection recommendations developed by the Enduring Guidelines effort, which will likely accelerate the adoption of HPV self‐collection in US cervical screening programs. Evaluating the use of self‐collection in screening programs will be important to quantify the extent to which self‐collection truly expands the screening coverage to underscreened populations and to understand how many screening participants prefer self‐collection over clinician collection for their HPV test. Cervical cancer is a major health problem worldwide, with the highest incidence and mortality in countries that currently do not have any screening. The ambitious cervical cancer elimination goals formulated by the World Health Organization require high coverage of HPV vaccination and cervical screening, which, in most places without existing screening programs, can only be achieved with widespread implementation of HPV self‐collection, allowing health workers to focus on those who screen positive.

## FUNDING INFORMATION

Intramural Research Program of the National Cancer Institute

## CONFLICT OF INTEREST STATEMENT

The authors declared no conflicts of interest.
